# Clinical evaluation and biological understanding of the early step-by-step healing after periodontal microsurgery: A case report with PES analysis comparing initial and 31-day result

**DOI:** 10.34172/japid.2022.016

**Published:** 2022-09-26

**Authors:** Sergio Kahn, Larissa Zarjitsky de Oliveira, Alexandra Tavares Dias, Gustavo Vicentis de Oliveira Fernandes

**Affiliations:** ^1^Veiga de Almeida University, Rio de Janeiro, Brazil; ^2^Department of Prosthodontics, State University of the Rio de Janeiro (UERJ), Rio de Janeiro, Brazil; ^3^Department of Periodontics and Oral Medicine, University of Michigan School of Dentistry, Ann Arbor, USA

**Keywords:** Assessment, case report, connective tissue, gingival recession, microsurgery

## Abstract

Microsurgery has evolved, permitting faster vascularization and healing than macro-interventions, reducing tissue trauma and obtaining precise wound closure. Therefore, this study aimed to detail the initial healing steps after the periodontal microsurgical procedure. A -26 year-old female had a localized recession (anterior lower tooth, recession type1-), with the absence of local keratinized tissue width (KTW) and adjacent gingival thickness (GT)<1 mm. After oral prophylaxis and occlusal adjustments, the pink esthetic score was performed (5 points), followed by the microsurgery procedure. Prior to inserting the subepithelial connective tissue graft (SCTG), the epithelial layer was removed, and the root surface was biomodified. Two days postoperatively, it was possible to observe a white layer from the SCTG in the gingival margin, decreasing after 4 days. In 6 days, the sutures were removed; no graft and volume loss was observed. For 9 days, the volume was the maintenance. Nevertheless, there was a reduction in tissue volume in the facial zone. After 11 and 13 days, an improved healing process was found, whereas, after 16 days, it was possible to report stable tissues, which was confirmed after 31 days, with a significant GR reduction and an increase in KTW and GT. Moreover, the final pink esthetic score (PES) was 9. Microsurgery had a faster healing and predictable outcome, suggesting reduced trauma, which may allow a quicker suture removal without jeopardizing the outcomes.

## Introduction

 Gingival recession (GR) is described as a root surface exposure to the oral environment due to the apical migration of the gingival margin relative to the cementoenamel junction.^1^The current treatment for this condition involves periodontal plastic surgery, improving esthetics, and preventing further progression.^2,3^ Many techniques have been described,^4^ including the coronally advanced flap (CAF) and subepithelial connective tissue graft (SCTG), preferred in terms of a better root coverage percentage and performance.^5^

 After surgical procedures, a typical response to the injuries will involve three overlapping and distinct stages: hemostasis and inflammation, new tissue formation, and remodeling.^6^ Pursuing to reduce the level of trauma and consequently the inflammatory profile, the concept of microsurgery has evolved, permitting a minimally invasive surgical protocol using optical magnification and special instruments.

 A recent study reported that flap design using microsurgical techniques positively impacted the outcome after root coverage (RC) procedure,^7^ providing a faster vascularization and a significant improvement in the healing process compared to macro-intervention.^8^ Also, reduced trauma and precise wound closure were reported. Otherwise, when performing micro procedures to collect SCTG, it tends to keep a greater quantity of epithelial layer on the connective tissue harvested (de-epithelized technique),^9^ which might impair the healing process and esthetic results.

 Moreover, there is a lack of a deeper understanding of early biological healing after surgical procedures, which might be explained by the complexities involved in the biological process and the clinical availability of the patient.^10^ Accordingly, there are other specific limitations, such as (i) a lack of assessment of all important parameters, (ii) articles and indexes that evaluate the healing one/two weeks after surgery (not since the beginning of the healing process), (iii) objective assessments without details, and (iv) the absence of calibration among surgeons and standardization of techniques (different kinds of incisions).

 Thus, this case report aimed to report the microsurgery procedure and detailed early steps of gingival healing after RC treatment, discussing the clinical outcomes with the biological knowledge.

## Case Report

###  Diagnosis

**Figure 1:**
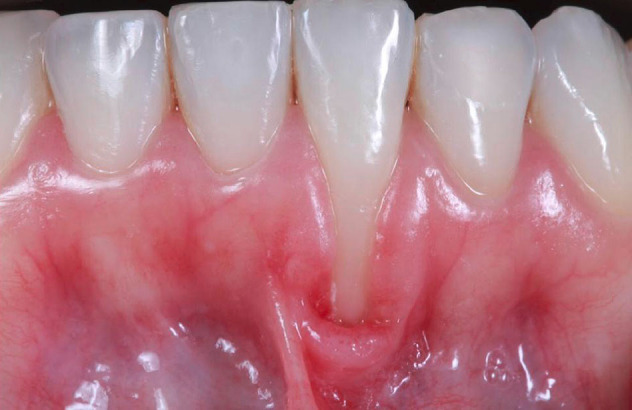
Initial frontal view of the gingival recession at tooth #24.

 A 26-year-old healthy female patient presented to the private dental office (Rio de Janeiro, Brazil) in May 2019 with a chief complaint of lower root exposure. A full periodontal screening was performed: (probing depth [PD] <4 mm, bleeding on probing [BOP] <10%, and plaque index ≤20%). Upon intraoral examination, a localized recession was noted on tooth #24 (ADA) or #31 (FDI) (lower right central incisor), with 7 mm of buccal recession and 2 mm of width (recession type 1, RT1),^11^ with no mobility and no interproximal bone loss (Figure 1). There was the absence of local keratinized tissue width (KTW) and the adjacent gingival thickness (GT) less than 1 mm. The goal of the surgical treatment was to reestablish the local gingiva, trying to augment the GT and KTW and prevent further progression of the recession.

###  Case management

**Figure 2:**
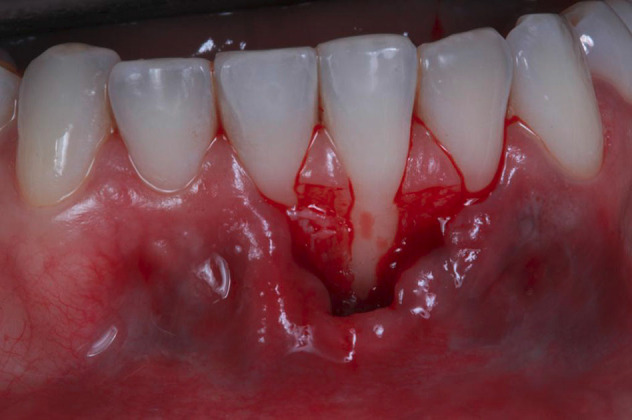
Horizontal incision at the papilla base with blade #15C.

 The patient received periodontal prophylaxis, occlusal adjustments, and hygiene instructions. Then, the first assessment for the pink esthetic score (PES) was performed, which achieved only 5 points out of 10 (Table 1). After infiltrative anesthesia (2% lidocaine, 1:100,000 epinephrine), the microsurgical technique was performed.^12^ Initially, horizontal incisions (#15C blade) were slightly coronal to the CEJ level at the mesial and distal papillae (Figure 2). Afterward, under ×8 magnification with a surgical binocular microscope (DV Vasconcelos), a split-thickness flap was initiated with a microsurgical blade (SB003 - MJK, Marseille, France), extending beyond the mucogingival junction.

**Table 1:** Pink esthetic score (PES) at baseline and after 31 days for tooth #24 (ADA)/#31 (FDI) (maximum achieved = 10)

	**Before surgery (day 0)**	**31 days after the procedure**
**Mesial papilla**	2	2
**Distal papilla **	2	2
**the curvature of facial mucosa**	0	2
**Level of facial mucosa**	0	1
**Root convexity/soft tissue color and texture**	1	2
**PES SCORE**	5	9

0 = absent or major discrepancy; 1 = incomplete or minor discrepancy; 2 = complete or no discrepancy.

**Figure 3:**
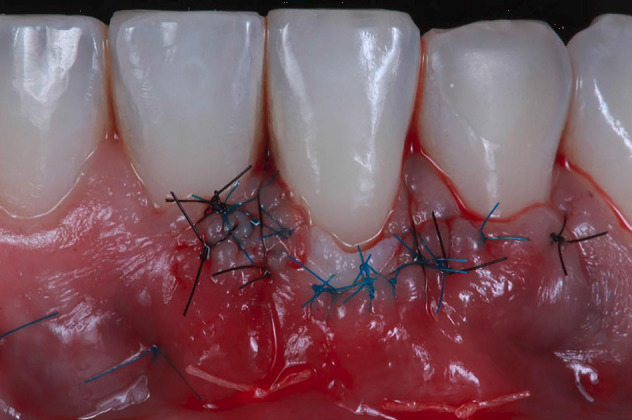
Sutures were performed to keep the SCTG and the flap in position.

 After measuring the receptor area, a mesiodistal (6 mm) SCTG was harvested from the palate at a 1-mm thickness using a double-blade scalped blade. The epithelial layer was removed during the preparation of the SCTG.^13^ Before the SCTG insertion (Figure 3), 24% EDTA (Straumann PrefGel, 24% EDTA, Straumann Group) was applied for root surface biomodification for 2 minutes, followed by applying enamel matrix proteins (Emdogain, Straumann Group, Switzerland).

 Then, the suture was performed in two steps. Firstly, an approximation suture with a 5/0 thread (Resotex-Resorba, Bayern, Germany) was used to position the flap at the papilla base. Secondly, for coaptation, a 7/0 (Resolon-Resorba, Bayern, Germany) interrupted suture was placed without trespassing the graft to guarantee an edge-to-edge position, involving the flap and SCTG to bed the tissues adequately, seeking to favor the vascularization of the graft. The patient received instructions and a course of treatment with 500-mg amoxicillin (tid) for seven days, 4-mg dexamethasone (bid) for three days, and 1-g dipyrone (qid) for five days orally. The sutures were removed six days postoperatively.

###  Clinical outcome

**Figure 4:**
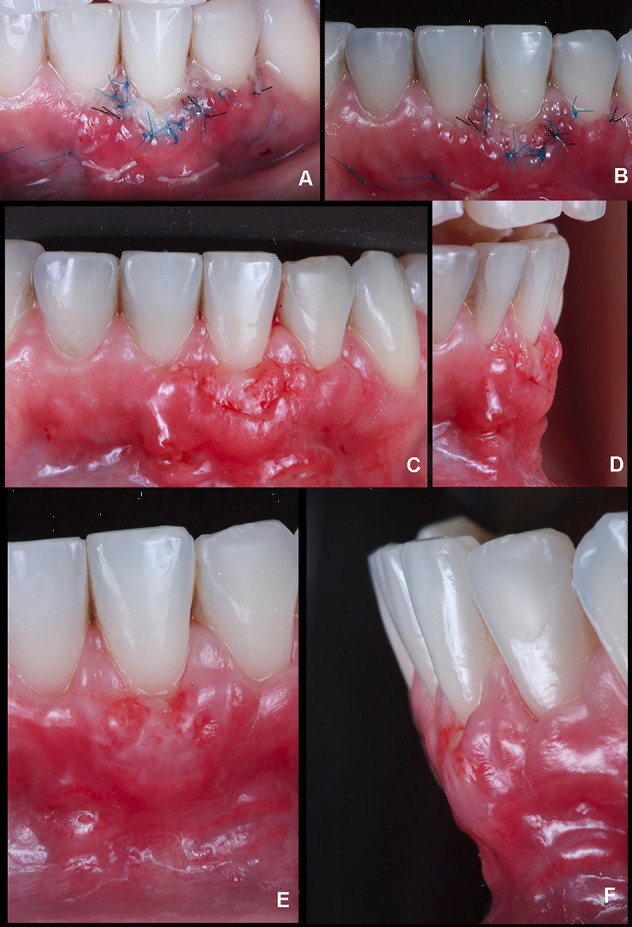
A. After two days, the initial healing with a white layer of the SCTG. B. After four days, a decrease in the white zone was evident, suggesting initial vascularization and adaptation of all tissues. C. Six days after the sutures were removed; the early healing phase. D. Lateral picture at six days, showing that the volume was preserved. E. Nine days after surgery, the frontal image shows the remodeled tissue. F. Lateral picture at nine days showing that the volume was gained.

 The patient was closely monitored to detail the postoperative period. Thereby, after two days, it was possible to observe a small white layer from the SCTG in the gingival margin (Figure 4A). In 4 days, there was a better tissue integration with a slight modification of the color on the white zone initially found (Figure 4B). After six days, when the sutures were removed, an interesting initial outcome was achieved, with stable tissue healing with no graft and volume loss (Figure 4C, D).

**Figure 5:**
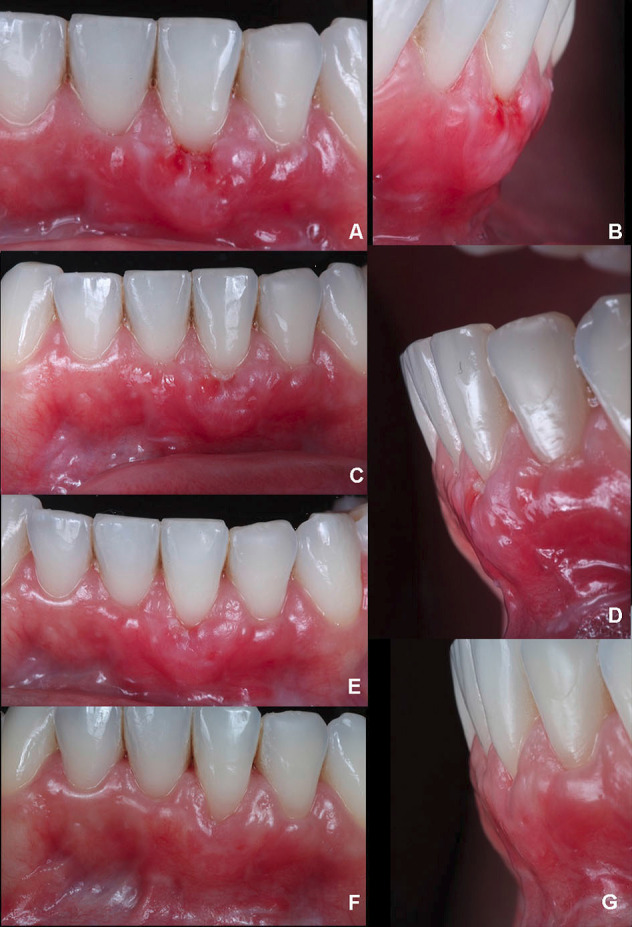
A and B. Eleven days after the procedure, C and D. Thirteen days after surgery. Volume was maintained, but a slight recession could be observed. E. After 16 days, the tissues stabilized. F and G. After 31 days; stable tissues, with normal coloration and exciting volume.

 Within nine days, a high level of vascularization and integration of the soft tissue graft was verified, reducing the white layer and maintaining the volume. Nevertheless, there was a reduction of tissue height in the facial zone (Figure 4E, F) compared to the outcome at six days. After 11 (Figure 5A, B) and 13 days (Figure 5C, D), improved healing was found with slight differences between them. In 16 days (Figure 5E), it was possible to report stable tissues, confirmed after 31 days (Figure 5F, G), with a significant GR reduction and increased KTW and GT. Moreover, it improved the PES analysis score from 5 (day 0) to 9 (31 days).

## Discussion

 The microsurgery approach brought a new perspective to the field of periodontal surgeries. Therefore, there is a limitation on knowing the details involving the initial steps of the healing and the biological process. Then, this case report aimed to give a more comprehensive understanding of this early stage, which is considered crucial to the success of the treatment.

**Figure 6:**
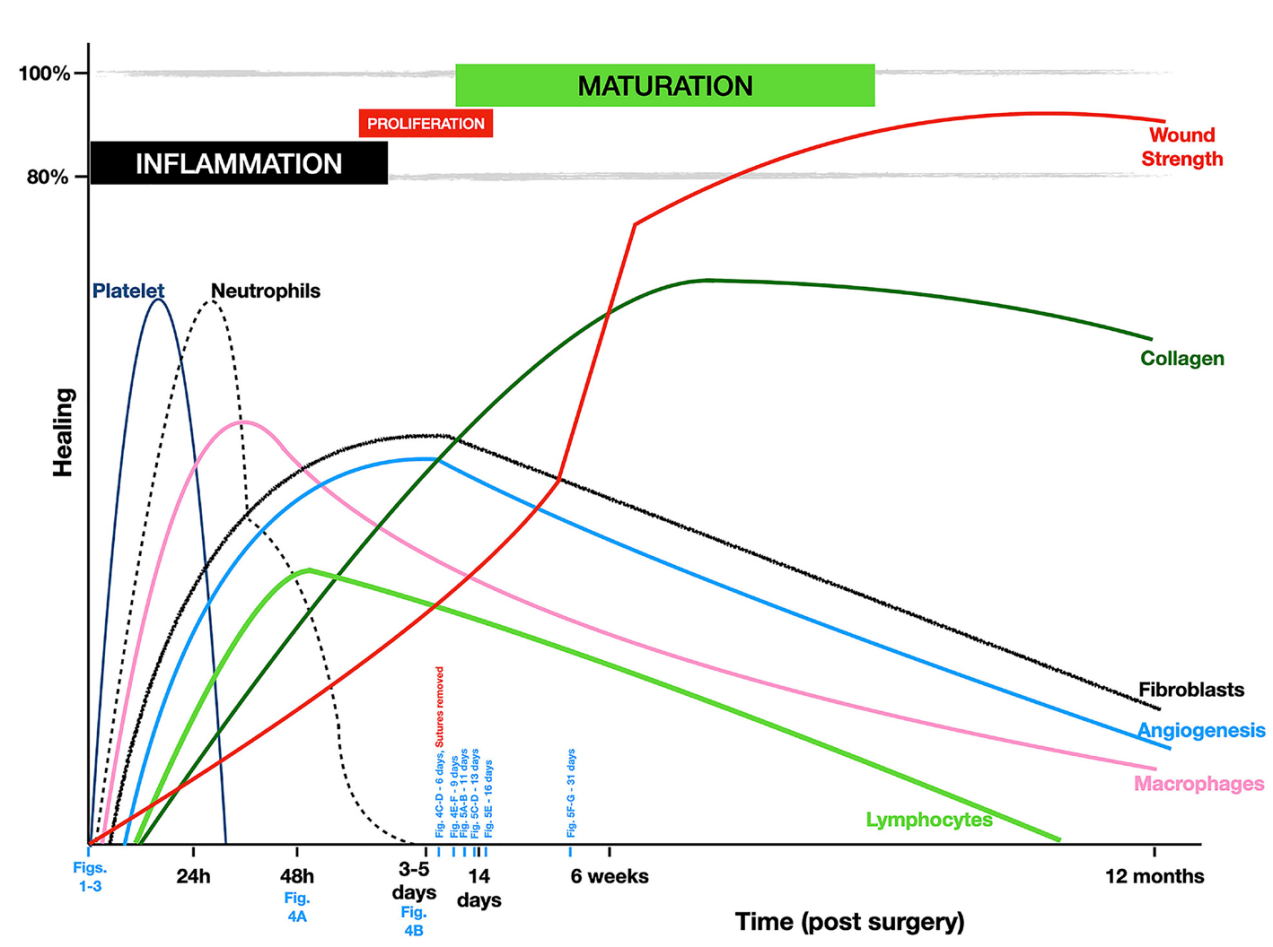
Biological and clinical behavior and inflammatory phases are compared graphiccally.

 Periodontal wound healing (Figure 6) begins with the presence of a blood clot, which will provide a provisional matrix for cells originating from the surrounding tissues.^14^ In the early inflammation stage (the first three days), while clinically, the tissues are undergoing the initial healing, the inflammatory cells (neutrophils, macrophages/monocytes, and lymphocytes) are attracted through chemotaxis.^15^In addition, collagen fibers, endothelial cells, and fibroblast populations proliferate into the wound area, permitting (after four days) initial integration and a local slight color modification.

 After six days, the sutures were removed. Although the literature shows that early suture removal (<10 days postoperatively) can negatively impact the attainable complete RC in single-tooth recession defects treated by CAF alone^16^ and may lead to the dehiscence of the gingival margin,^17^ precise technique following microsurgical principles has shown up to 30% higher complete RC than in macrosurgical approaches.^8^ The improved outcomes in this case may be attributed to vascularization and tissue adaptation, justified by understanding the healing process (as verified between 16 and 31 days after surgery). Consequently, throughout several phases of cell proliferation, matrix formation and repair (remodeling and maturation)^15^ might impact the removal of suture time, as observed.

 Moreover, it is essential to highlight the risk of necrosis, mainly associated with graft exposure, which lacks direct blood supply. Some sources of blood supply to an SCTG are interproximal bone, periodontal ligament, periosteum, and overlying flap.^18^ Thus, the most coronal part of the grafted tissue exhibits a complete absence of vessels, as observed in this case, resulting in local tissue loss and remodeling. Also, coronally advanced flap (CAF) without vertical incisions (envelope type of flap), associated with a subepithelial connective tissue graft, has resulted in a better outcome (stability and maintenance of the keratinized tissue width) than when used vertical tissue discharges after 3 and 7 years.^19^

 This case report had limitations, such as its case report nature; therefore, the data must be interpreted carefully. In addition, there were restrictions in the analysis, such as histological analysis.

## Conclusion

 It was possible to verify that microsurgery permitted a faster healing and predictable outcome, suggesting reduced trauma, which may allow a quicker suture removal without jeopardizing the outcomes.

## Acknowledgments

 None.

## Competing Interests

 The authors declare that they have no competing interests concerning the authorship and/or publication of this paper.

## Authors’ Contributions

 Conceptualization, SK; methodology, SK, ATD; validation, SK, LZO, ATD, GVOF; formal analysis, SK, GVOF; investigation, SK, LZO, ATD; resources, SK; data curation, SK, GVOF; writing—original draft preparation, GVOF; writing—review and editing, GVOF; visualization, SK, LZO, ATD, GVOF; supervision, SK; project administration, SK.

## Funding

 The authors received no financial support for the research, authorship, and/or publication of this article.

## Availability of data

 The datasets used and/or analyzed during the current study are available from the corresponding author on rea­sonable request.

## Ethics Approval

 None.

## References

[1] Agudio G, Cortellini P, Buti J, Pini Prato G (2016). Periodontal conditions of sites treated with gingival augmentation surgery compared with untreated contralateral homologous sites: an 18- to 35-year long-term study. J Periodontol.

[2] Cortellini P, Bissada NF (2018). Mucogingival conditions in the natural dentition: narrative review, case definitions, and diagnostic considerations. J Periodontol.

[3] Pini Prato G, Di Gianfilippo R (2021). On the value of the 2017 classification of phenotype and gingival recessions. J Periodontol.

[4] Roccuzzo M, Bunino M, Needleman I, Sanz M. Periodontal plastic surgery for treatment of localized gingival recessions: a systematic review. J Clin Periodontol 2002;29(Suppl 3):178-194. discussion 195-196. 10.1034/j.1600-051x.29.s3.11.x 12787218

[5] Rosetti EP, Marcantonio RA, Rossa Jr C, Chaves ES, Goissis G, Marcantonio Jr E (2000). Treatment of gingival recession: a comparative study between subepithelial connective tissue graft and guided tissue regeneration. J Periodontol.

[6] Gurtner GC, Werner S, Barrandon Y, Longaker MT (2008). Wound repair and regeneration. Nature.

[7] Chambrone L, Pini Prato GP (2019). Clinical insights about the evolution of root coverage procedures: the flap, the graft, and the surgery. J Periodontol.

[8] Burkhardt R, Lang NP (2005). Coverage of localized gingival recessions: comparison of micro- and macrosurgical techniques. J ClinPeriodontol.

[9] Kahn S, Araújo ITE, Dias AT, Souza AB, Chambrone L, Fernandes GVO (2021). Histologic and histomorphometric analysis of connective tissue grafts harvested by the parallel incision method: a pilot randomized controlled trial comparing macro- and microsurgical approaches. Quintessence Int.

[10] Marini L, Rojas MA, SahrmannP SahrmannP, AghazadaR AghazadaR, PilloniA PilloniA (2018). Early Wound Healing Score: a system to evaluate the early healing of periodontal soft tissue wounds. J Periodontal Implant Sci.

[11] Cairo F, Nieri M, Cincinelli S, Mervelt J, Pagliaro U (2011). The interproximal clinical attachment level to classify gingival recessions and predict root coverage outcomes: an explorative and reliability study. J Clin Periodontol.

[12] Campos GV, Bittencourt S, Sallum AW, Nociti Júnior FH, Sallum EA, Casati MZ (2006). Achieving primary closure and enhancing aesthetics withperiodontal microsurgery. PractProcedAesthet Dent.

[13] Maia VTG, Kahn S, Souza AB, Fernandes GVO (2021). Deepithelialized Connective Tissue Graft and the Remaining Epithelial Content After Harvesting by the Harris Technique: A Histological and Morphometrical Case Series. Clin Adv Periodontics.

[14] Wikesjo UM, Crigger M, Nilveus R, Selvig KA (1991). Early healing events at the dentin-connective tissue interface Light and transmission electron microscopy observations. J Periodontol.

[15] Susin C, Fiorini T, Lee J, de Stefano JA, Dickinson DP, Wikesjo UM (2015). Wound healing following surgical and regenerative periodontal therapy. Periodontol 2000.

[16] Tatakis DN, Chambrone L (2016). The Effect of Suturing Protocols on Coronally Advanced Flap Root-Coverage Outcomes: A Meta-Analysis. J Periodontol.

[17] Atterbury RA, Vazirani SJ (1961). Removal of sutures following oral surgery. Oral Surg Oral Med Oral Pathol.

[18] Lang NP, Lindhe J. Clinical Periodontology and Implant Dentistry. 6th ed. Chichester, West Sussex (UK): Wiley-Blackwell; 2015.

[19] Bhatavadekar NB, Gharpure AS, ChambroneL ChambroneL (2022). Long-term evaluation (7 years) of coronally advanced flap with (CAF) and without (e-CAF) vertical release incisions using a subepithelial connective tissue graft in the treatment of multiple recession-type defects. Quintessence Int.

